# Dwelling in Strangeness: Accounts of the Kingsley Hall Community, London (1965-1970), Established by R. D. Laing

**DOI:** 10.1007/s10912-020-09656-0

**Published:** 2020-08-26

**Authors:** Adrian Chapman

**Affiliations:** Florida State University London Centre, 99 Great Russell Street, London, WC1B 3LH UK

**Keywords:** R. D. Laing, Kingsley Hall, 1960s, Antipsychiatry, Asylum,, Counterculture, Countercultural psychiatry

## Abstract

This article explores archival accounts of the experimental community, Kingsley Hall (1965-70), established by R. D. Laing, the radical Scottish psychiatrist. The paper contributes to renewed interest in Kingsley Hall, R. D. Laing's radical psychiatry and UK counterculture. Archival sources enable not only the further exploration of already known figures but also let us hear previously unheard voices. Following a discussion of archival materials, the Hall is analyzed thematically and historically as (i) an inner spaceship; (ii) an embattled middle-class countercultural plantation; (iii) a site of spiritual renewal and development; (iv) a single-building arts colony; and (v) a countercultural experiment. Finally, it is argued that with re-evaluation of 1960s and 1970s counterculture now underway on the Left, the Hall’s experiment in Laingian countercultural psychiatry—as we may fittingly call it—may yet inform future radical projects (in mental health and beyond).

## Introduction

Here I focus mostly on unresearched archival materials associated with *Asylum: To Dwell in Strangeness*, an unpublished (and incomplete) book about Kingsley Hall (1965-70) and other communities in London, England, run under the auspices of the Philadelphia Association (PA), a mental health charity set up by the radical psychiatrist, R. D. Laing (1927-1989) in 1965. While there have been brief references to the *Asylum* archive (Harris 2012, McGeachan 2016), no one has yet drawn widely on the materials. A small number of documents have appeared on the internet, but most are in the Laing archive at the Special Collections Department, University of Glasgow Library. Documents include narratives written by visitors and residents, letters and poems, as well as transcriptions of audio-recordings. My primary focus is on written narratives—commissioned texts composed by those who assumed the authority to write publicly circulated accounts. Much of the *Asylum* material focuses on post-Kingsley Hall therapeutic communities established by Hall veterans. Detailed analysis of such material lies beyond my present scope, as (for the most part) does a consideration of interview transcripts.

This article contributes to renewed interest in Kingsley Hall (Fowler 2001; Harris 2012; Chapman 2014 and 2020; McGeachan, 2014a and 2016; Marmion 2015; Mullan and Moreton, 2017; Wall 2017). Such focus is very much bound up with attention to R. D. Laing, and here I add another perspective to the renaissance of interest in Laingian psychiatry. Scholarship drawing on Laing's archive has thus far resulted in studies of his development as a psychiatrist (Beveridge 2011), Laing and religion (Miller 2009, 2012) and the significance of space(s) in his thought and practice (McGeachan 2014, 2016). While I am concerned here with Laing and Kingsley Hall, my emphasis is more on the Laing *network*, people clustered around the famous psychiatrist and those living at, or who visited, the Hall. While Laing established the Hall, resided there initially and continued to visit until its closure, he had little involvement in the *Asylum* book project, only taking part in it towards his death and contributing little (a brief introductory section and smatterings of editorial work). Turning to the archival sources enables not only the further exploration of already known figures (such as Laing and Mary Barnes, the Hall's "star" resident) but also allows us, through decentering research into Laingian psychiatry, to hear voices of Hall residents and visitors previously unrecognized by scholarship.

*Mary Barnes: Two Accounts of a Journey through Madness* by Mary Barnes and her therapist Joseph Berke, both of whom lived at the Hall, has so far been our chief source on the household. This essay follows on from an article published in this journal about Barnes (Chapman 2020) that explores Laing’s conception of madness as a voyage, Barnes’ ‘journey’ and spiritual development, her becoming an artist, the Hall’s household politics, and difficulties encountered in the project of moving beyond the binary, hierarchical staff-patient opposition. One cannot argue with Laing’s comment (in his "Book Proposal"), though, that at the Hall “no psychiatric diagnosis [was] made or treatments given” (MS Laing L259/90).^1^ The household, neither part of the National Health Service nor a private hospital or half-way house, was founded to explore the liberatory possibilities of madness. Diagnosed schizophrenics lived there, but so did others with no diagnosis. The latter category included those in great distress and those not in significant distress but interested in the Laingian project. The Hall's ethos was that anyone, however *apparently* sane, might benefit from exploring his or her inner space—possibly to the point of what would usually be considered psychosis. One resident, we shall see, held down a steady teaching job and underwent a journey into inner space during a sustained period when a physical ailment kept her from her job. The Hall's aim was that without medication, ECT or physical restraint—but, crucially, with support from fellow residents—residents might live through, and benefit from, disturbed states rather than suppress them. As Laing puts it again in his "Book Proposal," “a person could go through his experience—go through it and not deny it, go through it and not around it” (MS Laing L259/90). Laing established the Hall to facilitate madness as a self-healing process; not breakdown, but, as he writes in *The Politics of Experience*, a break-through (1967, 110). There was no paid staff; no formal therapeutic program or requirements (such as therapy sessions or attendance at regular meetings); and no formal system of referral (usually, to gain admittance, you needed to know someone in the household or its extended network and be deemed acceptable by those living there).

Barnes was the first resident at what was a large former community center in London's East End. A former nurse and nurse educator who had been hospitalized for schizophrenia, tube-fed and confined in padded cells, she came to the Hall to experience fully the madness she felt had been denied her by hospital treatment. Barnes regressed to babyhood, and, after several years of extreme distress, sometimes punctuated by partial recovery, she emerged from her regression able to deal with formerly unbearable feelings of aggression and, finally, to begin differentiating her ego from that of others. The care of Joseph Berke, the young American psychiatrist with whom she went on to write *Two Accounts*, was vital to her recovery, as was her painting, begun at Berke’s suggestion. Her Christian faith, too, was central; indeed, she understood her madness as a redemptive journey bringing her closer to Christ (even though Berke understood her experience rather in psychoanalytic terms). *Two Accounts* presents a “success story”; but Barnes’ recovery, which takes place in the often-difficult atmosphere of the Hall (in which Mary comes into serious conflict with other residents and the Hall itself under attack from nearby residents), is long and arduous, involving periods when she is “up” and “down” again over five years. Her story contrasts with Laing’s implication in the *Politics of Experience* that madness might be a *brief*, recuperative, self-healing voyage—Laing gives the title “A Ten-Day Voyage” to a chapter detailing his sculptor friend Jesse Watkins' recuperative breakdown (see Chapman 2020 for a detailed discussion of Barnes at the Hall).

In the late 1960s and the 1970s, Barnes’ story attracted media attention. She became a radical psychiatric celebrity of interest to the press (Doyle 1968; Gillie 1969; Tyrer 1969) and featured in a play by the left-wing English writer, David Edgar (1979). *Two Accounts* was translated into thirteen languages, and most responses to the Hall have focused on Barnes. But she was just one resident in a large community—there was space for about a dozen people to have single rooms—that lasted five years. The *Asylum* papers (which include a Barnes contribution) contain several stories, analysis of which, building upon existing historical scholarship (Chapman 2020), open new understandings of the Hall. While Joseph Berke has underlined (Personal Communication) that there were many Kingsley Halls—each resident having his or her own perspective, or version, of the community—it is possible to draw out a number of thematic threads from archival accounts of Hall life.

The question I brought to the *Asylum* materials was “What do accounts tell us about the nature of the Hall?” How, in other words, can these hitherto largely unexplored accounts help us characterize the community? Following an account of *Asylum's* genesis, scope and intended audience, I analyze the Kingsley Hall as (i) an inner spaceship, a location for exploring the psychic interior; (ii) an embattled middle-class countercultural plantation in a working-class locale; (iii) a site of spiritual renewal and development; (iv) a single-building arts colony; and (v) a countercultural experiment with the accent on therapy as attention to the practice of everyday life.

My approach takes archival sources as *starting points* for discussion and thematization. Following the account of the *Asylum* book project, each section refers to archival material, historically contextualizes this material and interrogates the nature of Laingian psychiatry. The section on the Hall as countercultural experiment (which considers the meanings of "research" and "experimentation") particularly broadens discussion beyond *Asylum* materials. In the conclusion, I consider the Hall as a utopian project of possible current and future value.

## *Asylum*: *To dwell in strangeness*: The book project

The project began in summer 1969 after Laing asked his colleague and fellow Hall resident, the US-born Dr. Leon Redler, to edit a book on life at the Hall. Another resident, Paul Zeal, a recent graduate of Bristol University and The Institute of Education, worked as Redler's editorial assistant. Later Michael Yocum, an American who also lived at the Hall, became a second associate editor. The Hall trustees’ 1969 decision not to renew the PA lease delayed the book's completion as did lack of clarity about the project's nature (according to Zeal in his "Ideas Towards a Preface," MS Laing L221/10). Originally, the book was to include first-hand accounts of the Hall by residents and visitors, and PA network members, plus edited transcripts of interviews with residents and local East End residents and community members. As the project fitfully continued, the book expanded to include sections on later PA communities based in London (in Archway, Holland Road and Portland Road). Over time, though, the project narrowed in focus, the emphasis falling on accounts written by residents of, and visitors to, communities. Archival holdings are in the Special Collections department at the University of Glasgow Library.

While there was a book contract with the UK publisher, Tavistock, and a US publisher, the book never made it into print (Redler, Personal Communication). The publishers, though, must have seen the market potential in the book. Key to its appeal would have been the association with Laing who was in the late 1960s and 1970s the most famous psychiatrist in the world, a celebrity and an icon of the counterculture. His books became part of the countercultural "canon,” essential reading for the young radical. *Asylum* would have offered inside views of Kingsley Hall and other therapeutic communities, complementing *Two Accounts.* With Laing's imprimatur, *Asylum* would likely have sold to readers who identified with the counterculture, offering insight into not just radical psychiatry but also the Hall as a practical countercultural experiment. Like Berke’s 1969 edited volume, *Counter Culture: The Creation of an Alternative Society, Asylum* would have offered accounts of quite what the "alternative society" was like in practice. People who might never have visited an anti-psychiatric community or never consulted a psychiatrist, let alone one of the few Laingian psychiatrists, could have read the book and, perhaps, felt a kinship with a psychotherapeutic countercultural project.

In the 1980s, Redler decided to resurrect the project and asked Laing if he would come on board as an editor. Laing died in 1989, however, not having accomplished any substantial editorial work and having written only some brief introductory material. The last documents associated with the book are from 1983. Laing's star had faded by the 1980s, the age of large mental hospital closures, “community care” and widespread use of new psychiatric drugs. Nevertheless, had the text reached bookstores in the 1980s or 1990s, the Laingian association would have lent it significant appeal, long after the closure of the Kingsley Hall project.

The book’s most settled title was *Asylum: To Dwell in Strangeness.* Other rejected titles were *To Dwell in Strangeness: Studies in Alien States of Mind* and *The Open Way*. The phrase “To Dwell in Strangeness” came to Redler’s notice through Keith Musgrove, an Archway resident who contributed a brief essay entitled “To Live in Strangeness” (MS Laing L1771). Musgrove alludes to Samuel Beckett’s *Malone Dies*: “A bright light is not necessary, a taper is all one needs to live in strangeness, if it faithfully burns" (1956, 182). The *Asylum* book would have provided a guide to the Hall and other communities by holding up a candle (“a taper”) to its “strangeness.” We can think of strangeness as a peculiarity, deviance from the norm; in my discussion, it will be evident how the Hall was in several respects strange in this way. We can also think of strangeness in terms of *estrangement*, which might concern distance from something or somebody or estrangement in the sense of defamiliarization, a sharper awareness of that which is ordinarily taken for granted (see Baldrick 2015). Both meanings of estrangement, we shall see, are relevant to the Hall. 'To Dwell,' which refers to residing somewhere, lends an archaic or literary quality to the Hall project. The phrase is also very close to "to dwell upon" with its suggestion of lingering with and paying attention to someone or something. We shall we see that the Hall had a strong artistic dimension and that paying sustained attention to the way in which one dwelt was very important there.

The University of Glasgow Special Collections Hall material that I focus upon here (with call numbers given in brackets) consist of notes from one of the editors (Paul Zeal); a book proposal and introduction by Laing; another introduction by Leon Redler; residents' accounts of community life comprising six prose texts and some poems composed by one resident; and three visitors' accounts of the Hall. I draw further on archive materials: a letter by Laing to someone who came to reside at the Hall; an interview with a doctor whose surgery was opposite the Hall; and two talks given by Laing during his 1972 US lecture tour. In the essay's final section, reference to an underground newspaper (*Friends*), available at UCL Library's Special Collections, helps me develop discussion of the Hall as an experiment. Sources' call numbers are given in parentheses in the text below.

## An inner-spaceship

Frances Horn Williams was living at the Hall while working as a schoolteacher in South East London. In “Bringing Home the Evening Sky,” she writes of vast differences between the school world, governed by the bell, and the more fluid time of the Hall, a household that woke up at night. A bout of thrombosis afforded her two and a half months’ leave from teaching, and during this time, she says, she underwent an experience that was “like a kind of death—a visit to the Underworld”:

I lost all sense of ‘I’, lost my ego. Why was I, and why are others, so afraid of doing this? It’s a freedom and release—a death that is a source of energy and strength.

A new beginning. Remember. As an infant again I remembered the first infancy: as a child again, my first childhood. This time was not like the last. I remembered and no-one taught me to forget. I learnt to play as a child as I had never played as a child and I brought back with me to the adult world the wonder of being.

I was lucky - I had a second chance. It has made a difference to every moment of my life, sleeping and waking, since. How is it that such a journey can derided sic]—‘withdrawing from your responsibilities’? (MS Laing L176/3)

Like Mary Barnes, then, Williams underwent a positively transformative regression (even if this would have been frightful to most people). Dwelling at the Hall, she became strange to her conventional, schoolteacher self, who "died." Like Barnes, she returned to childhood, and like Barnes, who details the games (e.g. playing at being a crocodile) she would play with her therapist, Williams learned to play again. Similarly as Barnes presents regression as fundamentally revivifying, Williams returns to the adult world a changed, and she tells us, enriched woman. But Williams' narrative covers three months, rather than the five years of Barnes' story (see Chapman 2020.) Williams’ testimony is the closest we come in Hall archival accounts to Jesse Watkins’ story, given by Laing in *The Politics of Experience* (chapter 7). Watkins, a sculptor and former mariner, descended into madness and re-emerged into everyday life in just ten days. Madness for Watkins involved experiencing time going backward, the stations of the cross and long chains of association set off by reading. Williams' story is not as spectacular yet if published would have testified to the tremendous advantages of a brief period of radical withdrawal and regression. With her writing about the death of her ego and rediscovery of childhood and re-entry into adulthood, we can view her (to use a phrase of Laing’s friend, the Scottish Beat writer Alexander Trocchi) as a “cosmonaut of interior space” (Bartie and Bell 2012, 108).

Alongside Mary Barnes' story, Francis Horn Williams' text is the only written female account of a transformative Hall journey. We have no figures on the Hall gender balance, but undated figures referring to several (unspecified) Laingian communities reveal a greater number of male residents who stayed in households for longer than three days: 273 men to 185 women (MS Laing L221/24). Noel Cobb, who lived at the Hall for almost two years, states that women found it difficult to settle there (MS Laing 169/2). The US psychiatrist Ross Speck, who visited, notes that women appeared marginal at the dinner Table (MS Laing 179/1). Barnes' story comes in the main from *Two Accounts* where she very much asserts her independence from her carer and co-author, Joseph Berke (see Chapman 2020). Yet the paucity of female Hall resident accounts in *Asylum,* together with Cobb's and Speck's observations, suggests the undervaluing of women in the community.

The Hall was a place for people—principally men, it seems—to traverse uncharted inner regions: a portal, a point of entry into unexplored psychic zones. Such zones, Laing argues in *The Politics of Experience*, are more distant than great mountain tops or even the vast reaches of outer space. This distance indicates the extent of post-war humanity's alienation from itself. In such circumstances, cosmonauts of inner space (like Williams) become pioneers and their narratives both signposts, and invitations to, the reclamation and enrichment of experience. To overcome alienation (according to Laing), it becomes necessary to “travel” into territory regarded conventionally as alien to normality, common sense and sound mental health. The ego, adapted to societal mores, must be “lost” (as was the case for Williams), allowing the possibility of more fluid, open and playful experience. In Laingian terms, Williams' story is one of *metanoia*, a term Laing probably found in Jung’s work and which Laing used to signify a “change of mind” (1972). The Hall, he believed, could provide a metanoic context.

In the community, one way of changing one’s mind—or, to employ the 1960s countercultural idiom, "expanding one’s consciousness"—was to take hallucinogenic drugs. In “Metanoia: Some Experiences at Kingsley Hall” (1972), Laing compares psychosis to the LSD experience, another way of traveling through inner space. While LSD was used at the Hall, the *Asylum* papers do not contain any records of drug experiences, perhaps because it became illegal in the UK in 1968 and the editor(s) did not want to associate the household with criminality. But Laing was one of the few doctors licensed to prescribe LSD (Robertson 1999, 59), and Berke has recalled LSD use at the Hall (Levy 2017). No one who took it, according to Berke, was any the worse for it; and for several people it was a very positive experience. A ground floor room set aside as a meditation space became the LSD room (Personal Communication). In his Kingsley Hall diary, Zeal (2008) writes of taking acid in the room. For him, the LSD experience was very much about the expansion of experience and spiritual development, closer to Aldous Huxley’s vision in his *Doors of Perception* than to hedonistic hippies taking drugs for “kicks.” LSD was taken for therapeutic self-discovery but not in the context of formal therapy. We might reasonably speculate that acid provided “rocket fuel” for at least *some* at the Hall to undergo inner space exploration. We are a long way, certainly, from formal experiments in LSD therapy carried out in the 1950s and 1960s or later experiments forming part of the “psychedelic renaissance” (see Dyck 2008).

Just as the metaphors of the “voyage” and “journey” to describe psychosis elevate madness, so the status of psychosis (or a similar, short-term drug-induced experience) is raised by viewing it as a means of traversing inner space. While Laing and his colleagues such as Berke and Cooper never denied that what was usually understood as psychosis could be extremely disturbing—unsettlingly strange, indeed—to the individual undergoing it and to others around him or her, such language takes us far away from pathology and the management of symptoms and rather encourages us to view psychosis as *potentially* valuable.

## An embattled middle-class countercultural plantation in a working-class locale

We encounter the term "inner space" in a poem written by Francis Gillett, a Hall resident, who described himself on a BBC radio program as a paranoid schizophrenic who embraced the possibility of going mad offered him by Laing. In an untitled Kingsley Hall poem, one of several that he wrote in 1985 (all untitled with pages unnumbered) and gave to Laing for the *Asylum* book, he relates inner space to the space immediately beyond the community’s threshold. He recalls the building being under siege by locals. The poem opens with the information that “A gang of thugs / Were hanging around outside.” (Ian, mentioned in the lines below, was a long-term resident, who went on to become a clothes designer. The “Mary” below is Mary Barnes.)

Ian secured the front door with a huge chain on the inside

And Mary stood in front of it

With her arms outstretched, crucified,

Begging us not to pass outside.

Gillett continues by emphasizing that " 'Inside' and 'outside' became an important issue" for him as "The boundaries began shifting." He goes on:

The concept of “inner space” was on everyone’s lips.

That it was as vast as the “outer” kind

Was quite clear to me,

I could see it stretching away infinite

when I closed my eyes to sleep.

And the music of trains and cars passing

Was like static in a radio telescope. (MS Laing L 232)

The term “inner space” is introduced after we are told that the community is under attack from outside. At the poem's close, the external world is registered only as a light-years-away scratchy noise (“static in a radio telescope”). Here the Hall is presented not simply as an ante-chamber, a point of departure for voyages into inner space, but rather a place under threat, and inner space, quite understandably, serves as a retreat from the hostility of violent neighbors.

Why were the locals so hostile? The *Asylum* papers provide some answers. In one document, the local London East End, Bromley-by-Bow, general practitioner (family doctor) speaks to Leon Redler and Paul Zeal of the difficulties locals had with the Hall, relating problems very much to the differences in social composition between the Hall and the surrounding area. Recalling his patients’ relations with the Hall, he makes clear that house-proud, local working-class people suspicious of "ne’er do wells" were disturbed by people with long hair and an unkempt appearance coming and going at all hours to and from the community, which they associated with drug-taking and parties. The household could have done more, the doctor claims, to build links with the medical authorities in the locality and more to inform the neighborhood as to what kind of household they had in their midst (MS Laing 206/1-2). When the building first came under PA occupation, there was a brief period when the new residents shared the space with local groups, but this soon changed. As Zeal notes in his diary: “Before the present set up, boy scouts and others used to use the basement, but now these have fallen away and now we have art exhibitions" (2008, 22). This sounds very much like gentrification, a term used today to describe the influx of relatively affluent, fashionable residents into low-rent areas. This process, which often results in the displacement of long-standing communities, can meet with considerable resistance from locals. While we might think of gentrification as a characteristically neoliberal phenomenon, the term was coined in 1964 to describe the taking over of working-class London by middle-class newcomers (Glass 2010; Lees et al. 2010). At a time when the East End of London was still scarred by the war and considerable numbers of residents were departing to nearby new towns, the cosmonauts of inner space at the Hall may have seemed like the harbingers of fundamental and unsettling cultural and economic change. In fact, the new Hall residents did not turn out to be the advance guard of gentrification that led directly to the displacement of local, poorer people, but fear of this may well have been in play.

Outsiders not only moved into a culturally distinct area, but they also took over a building important to the neighborhood. Strangers, peculiar to the locals, occupied a significant community dwelling. The Hall opened as a pre-welfare state community center in the 1920s. By the 1960s, it had fallen into disuse and was rented to the PA at a nominal rate. But it had been a thriving local institution hosting lunch clubs, youth clubs, classes, nurseries, clinics, legal advice, and debating societies. In 1931, locals flocked to the Hall to see Mahatma Gandhi, who stayed there for several months when attending the Roundtable Conference on the Independence of India. Hiroshima-Auschwitz peace marchers stayed at the Hall in 1963. The building, then, had been very much a part of the local community and linked Bromley-by-Bow in the East End with wider progressive causes and concerns. Then came the Philadelphia Association. Today the Kinsgley Hall Community Centre official website states that "Unfortunately, given habits of residents in the 'no-holds-barred ' experiment, to howl at night or walk into local pubs and finish all drinks on the table, the local community was largely hostile to the Philadelphia project." These words come in a section about the re-opening of the Hall entitled "Rehabilitation," a term that suggests the PA inflicted shame, disgrace or perhaps even “traumatic” injury on the building, a much-loved local institution.

There is just one archival record of a local person joining the inner space adventurers. Known as the “Jesus Man,” there is a reference to him in "Bedrock," an account of the Hall written by Noel Cobb, an American who moved into the community after having been in touch with Laing by letter and who would later become a psychotherapist and poet. The Jesus Man believed that God had spoken to him in a dream, instructing him to tend his flock. This he found at Kingsley Hall, where the largely middle-class residents were amazed that a young man from the East End— “a red-haired cockney boy”—could sing and play the guitar so compellingly. Cobb writes: “The earthy, hypnotic rhythms and the unrestrained, wild naivety charmed everyone. Most of us were startled to hear these words coming from such a funky, folky fellow straight out of the back-streets of London’s East End.” The Jesus Man sang: “I’ve discovered who I really am. I’m your son—I’m Jesus-Man. I know you know, you know I know I know you know you know I know” (MS Laing L169/2).

He treated the other residents as his disciples but did not pay his rent, became aggressive when others failed to play along with his fantasy, and was eventually required to leave. After his departure, according to Cobb, the household became increasingly suspicious of outsiders. I do not want to suggest that the Jesus Man was not extremely difficult to live with nor that the household was necessarily wrong in expelling him. But Cobb’s presentation of the Jesus Man sounds rather like an old-fashioned, moneyed white music critic discussing a poor black bluesman. The Jesus Man is “earthy” and “unrestrained,” with a charming "wild naivety." The language expresses admiration but patronizes, indicating the profound difference between the household, surprised by the “funky, folky fellow” and the denizens of East End London living only next door. We can relate this gap to the distance between much of the London “underground” in the 1960s and the working class. The division was noted sharply in the London-based 1960s underground magazine *Oz* by the left-wing activist and journalist, David Widgery (1989, 7-13). While he does not mention Kingsley Hall, he mentions “anti-universities,” and the London Anti-University, a radical education initiative closely connected to the Laing network, met for a time at the Hall. For Widgery, radical projects cannot hope to be genuinely transformative or have much value at all when abstracted from the daily lives and struggles of working people. This view resonates with the satirical treatment of the Hall in its one-time resident Clancy Sigal’s novel, *Zone of the Interior.* Neighbors attend an open day at "Meditation Manor" (Sigal's name for his fictionalized Kingsley Hall). Far from being impressed, they find the residents ridiculous, especially the “star” of the household, a former lawyer who, determined to realize her uniqueness by becoming a mermaid, spends her time in a large water tank. (The character is a send-up of Mary Barnes, the real Hall’s “star” resident.)

Speaking at a conference in 1981 at the University of Leuven, Belgium, David Cooper criticized PA households for being "islands" of privilege. Cooper was Laing’s one-time collaborator; both psychiatrists, the two wrote a book together, *Reason and Violence*. Before the Hall's opening, Cooper had attempted to transform a state hospital’s adolescent male wing (Villa 21, Shenley Hospital, St Albans, Hertfordshire) into a democratic community. Never much interested in the Hall, Cooper left the PA in 1971 protesting what he understood as the reactionary political drift of the organization (away from politics and towards mysticism). His words at the conference touched Laing because not only does he refer to them in his “Preface-Introduction” to *Asylum* (MS Laing 221/15A) but he also handwrites them (with no accompanying text) on a single sheet of (uncatalogued) paper: "What sense does it make to create ten happy islands in a world where everything keeps functioning just like before? In this way the institution is not being attacked. Madness is being recuperated, encapsulated by the system and loses its function to subversive activity" (for more on Cooper, see Chapman 2016).

Laing’s response is simply to argue that despite Cooper’s political concerns, people still break down and still require refuges such as the Hall and subsequent PA communities. We do not have a record of Cooper’s entire speech, but his published work, especially *The Language of Madness*, provides clues to how he may have elaborated his argument. He came to see PA households as bourgeois enclaves isolated from the wider community. Moreover, for Cooper, influenced by Franco Basaglia and the Italian *Psichiatria Democratica* movement (see Foot 2015), therapy was a political struggle in which individual symptoms, and family disturbances, were to be understood and confronted in broader contexts. This view was shared by Peter Sedgwick, a left-wing critic of Laing, who attacked Laing in the 1982 *Psycho-Politics.* For both Cooper and Sedgwick, therapy was indissoluble from socialist struggle and community work.

The Hall certainly became estranged from its neighbors, and class differences were a significant part of the neighborhood's antagonism. But the community was far from being an “island,” and we can view it as a political venture connected to key sites and events in countercultural London. Community members attended the 1965 International Poetry Incarnation at the Royal Albert Hall. This event, regarded by some as the inaugural event of the London underground, at which the US poets Allen Ginsberg and Lawrence Ferlinghetti spoke along with Alexander Trocchi and Adrian Mitchell from the UK, brought thousands of people together in what was, and still is, the largest ever poetry event in the UK (see Green 1998). Laing, Berke and Redler put on the Dialectics of Liberation in summer 1967, a two-week London event at which Ginsberg, the philosopher Herbert Marcuse, and the US black rights campaigner Stokely Carmichael spoke (see Cooper 1968). Along with Berke and Cooper, Laing participated in the London Anti-University, an educational initiative geared towards personal and social development and which began in 1968 and continued into the early 1970s. We can understand the Hall (which included residents from the UK, mainland Europe, Southern Africa and the USA), then, as a node in a countercultural network stretching across London and, indeed, across mainland Europe and the Atlantic to the States and beyond. The Hall's true "neighbors," then, were not the ones living next door but rather those who identified with the "the revolution,” wherever they may have been.

## A site of spiritual renewal and discovery

Speaking on BBC radio's "Saturday Live" in 2013, Francis Gillet remarked that he and the other residents were like early Christians finding asylum from the world’s hostility. In one of his 1985 Kingsley Hall poems, he writes:

I have no ideas about God or Jesus,

But suddenly, here I am in The Catacomb with these ‘Christians.’

Is it true then, are the rumours I have heard

Of a new way of life, a new world appearing, completely true? (MS Laing L232)

Here the Hall is associated with “a new way of life, a new world,” implicitly a different life from that of its neighbors. The Hall is a place of faith, an underground retreat (a “Catacomb”). The reference to the world of the dead associates the poem with the words of Williams, who underwent a voyage into “the Underworld” at the Hall and, like Mary Barnes, was then “re-born.” For some, the Hall was a site of spiritual renewal and discovery.

Barnes’ *Asylum* contribution underlines the religiosity she discusses in *Two Accounts*, where she makes clear her desire to lose herself in the Father and writes, “May all be shattered into God” (1971, 17). Privations suffered during her period of regression were, for her, a form of spiritual discipline. When she began to paint, her artwork had mostly religious themes, and through her madness, she deepened her (Catholic) faith. Her contribution to the book about PA communities is brief, a collection of loose notes, but she makes clear her spiritual focus. The text’s first section has paragraphs that open “I have changed,” “I have learnt,” “I have joy in sharing,” “I’ve learned to *live”* and, opening the climax of the section, “I thank God for my life” (MS Laing L178/1-5). While her therapist, Berke, did not share her spiritual focus and understood her regression in primarily psychoanalytic rather than spiritual terms, Barnes is close to Laing in viewing therapeutic progress and spirituality as inter-infused.

In *The Politics of Experience*, Laing argues that the problem for post-war Western humanity is not in essence one of repression but rather atrophied modalities of experience. Sense experience, dreaming, proprioception, fantasy, and spiritual experience had, according to Laing, become shrunken, foreclosing the possibility of full, authentic life. Voyaging through madness—exploring inner space—contains the possibility, however, of revivifying spiritual life and other modalities of experience through drawing on the wellspring that feeds all religion. In his perennialism, Laing echoes Aldous Huxley in his *The Perennial Philosophy*, in which he argues that all religions draw from and express a common source. In drawing from this wellspring, the mad person (who like Williams undergoes something very much like a conversion experience, or, like Barnes has her faith renewed) becomes in Laing’s language the “hierophant of the sacred” (1967, 109), a priestly figure with knowledge of great mysteries.

The Hall was the first of the Philadelphia Association communities. “Philadelphia” is the city of brotherly love; it is also one of the seven Asia Minor churches mentioned in Revelation. The PA motto comes from Revelations 3:8: “I know thy works: behold, I have set before thee an open door, and no man can shut it: for thou hast a little strength, and hast kept my word, and hast not denied my name” (King James Bible Online). Here the Philadelphian church is divinely commended for its steadfast belief despite its members’ weakness, while others have denied God. The choice of motto not only sanctifies the PA and the Hall project but also encourages us to understand the community as an outpost of faith surrounded by hostile unbelievers. Discussing the idea of asylum (to Laing always a "safe space," a sanctuary) at the University of California, Berkeley, on his 1972 US lecture tour, Laing said that the first therapeutic communities were Christian settlements that existed just after Christ's death. The Therapeutae, whom he presents as the first, and true, therapists, “regarded themselves as expressing their ideal of human relationships by being attendants upon one another, being at the service of each other, being attentive to or waiting upon [each other]" (MS Laing WA 41-42). Laing may in part have been informed by a history of the early church by Eusebius of Caesarea (1998), who suggested that the Therapeutae, a Jewish sect about whom Philo of Alexander wrote, were the model for early Christian communities. There does not seem to be any historical evidence that the Therapeutae, an ascetic sect who practiced healing and whose members lived largely in isolation from one another, were anything like proto-Laingian therapists. Nevertheless, it is noteworthy and not at all surprising that Laing aligned his work in community psychiatry with early Christian communities. Laing was, as Gavin Miller points out, someone for whom madness represents "the lost essence of early Christian experience" and "[t]he role of the therapist is therefore to assist in this conversion or rebirth" (2009, 15).

Laing's interest in "the essence of early Christian experience" is apparent again in his choice of epigraph for his prose poem, *The Bird of Paradise.* He chose an extract from the Gospel of Thomas, a so-called Gnostic gospel, one of several representing a current of early Christianity that were discovered near Nag Hammadi, Egypt, in the mid-1950s. The extract emphasizes the need to overcome artificial, worldly dualities to be at one with Christ. The fascination with early Christianity was not restricted to Laingians alone, however. The Jung institute purchased a volume of the Nag Hammadi library in 1951, and the Jung Codex formed the basis of the gospels' first (partial) translation in 1956. Beyond the world of psychotherapy, early Biblical Christianity influenced new religious movements; for instance, the counterculture-inflected Jesus People Movement (JPM). The JPM offered a born-again form of discipleship characterized most visibly by contemporary-style worship including guitars and drums and street "witnessing," disciples publicly avowing their faith. The movement found Biblical authority for communal living in The Acts of the Apostles, in which Luke presents the early Jerusalem church (Acts 2:42-47 and Acts 4:32-37). The Jesus People held some common ground with the early charismatic movement into which JPM members often drifted. The charismatics, themselves often interested in communal living, emphasized the lives of Jesus' disciples for whom the spirit was still abroad (see Harper 1973; Walker 1988). Similarly to Laing, charismatic Christians understand faith as an embodied experience. (Laing reminds his *Politics of Experience* readers that Saul of Tarsus was struck down by his encounter with God.) Francis Gillett's characterization of Hall residents as Christians, then, should be understood as part of a much wider interest in both canonical and non-canonical early Christianity, an interest played out in both mainstream culture and counterculture.

Spirituality at the Hall was not exclusively Christian, however. Some residents, including Laing, practiced meditation and yoga. (Not long after the Hall's closure, Laing would spend a year-long sabbatical consisting of periods in a Sri Lankan Buddhist temple and with a holy man on the foothills of the Himalayas.) Both Zeal and Yocum write of the Hall as a place in which the conventional self might be transcended and a more enlightened, authentic and spiritual self emerge. For Zeal, “Self-attachment, clinging to self, is perhaps the first sign of madness, based upon cut-off within the great stream of life” (MS Laing L218); for Yocum (MS Laing L184/1-2), the Hall enabled estrangement from conventional bearings such that he was able to better appreciate the mindful state of being in the moment. Whether they adopted an Eastern or Christian approach to spiritual development, Yocum, Zeal, and Barnes understood the Hall as a place in which an alienated ego, adapted to conventional mores, might melt away in favor of a more authentic, satisfying and spiritually infused self—the defining features of New Age “Self-Spirituality” set out by Paul Heelas (1996, 18-20). For Barnes and Williams, this elevated state was attained through journeying into inner space; for Zeal and Yocum it was more a matter of living in the community and allowing their presuppositions to be challenged. For all four, the Hall’s therapeutic nature was indivisible from it being a site of spiritual renewal and development. Estranged from bourgeois convention, the self could come "home" and dwell upon itself and the world differently.

## A single-building art colony

Mary Barnes’ paintings were almost exclusively religious in theme. She repeatedly painted the resurrection, not surprisingly as she felt she was undergoing a spiritual trial involving death and rebirth. But Barnes was not the only artist in the household, which was a place of considerable cultural production; so much so that we can consider it a single-building art colony.

The South African artist, Harry Trevor, lived at the Hall, and the painter Felix Topolski and sculptor Jesse Watkins visited and encouraged Barnes in her work. There were also numerous writers at the Hall. The American novelist, Alfred Chester, spent most of his time in the Hall working alone in his room on the proofs of his novel, *The Exquisite Corpse*. The Norwegian writer, Axel Jenson, a friend of fellow Hall resident and poet, Noel Cobb, lived there; as did Clancy Sigal, the US left-wing activist and journalist, who later wrote *Zone of the Interior*, a r*oman à clef* concerning the community. The African-American poet and sociologist, Calvin Hernton, lived in the household. Julian Beck, the founder of the radical theatrical company, The Living Theatre, visited and lectured, as did the British playwright, David Mercer. Berke wrote a play that was performed at the Hall and edited a literary/countercultural magazine, *Fire*, which featured several Hall residents and Laing network members. (The three issues of *Fire* can be viewed in The British Library.) Of these persons, only Cobb (with his essay on Hall life, MS Laing L169/2) is represented in the *Asylum* papers. Berke split from Laing and the PA around the time the Hall project ended in 1970, but *Asylum* would certainly have represented something of the household's arts orientation.

The book would also have featured several genres and points of view. Originally, Redler hoped to include not just words but also images. The only images among the documents, though, are marginal drawings in a letter to her son by the American-Indian psychologist, Carolyn Attneave (Uncatalogued). Struggling for words to express the Hall's nature, something unusual, bizarre and exciting to her, she includes drawings of the building and residents. *Asylum* documents include Francis Gillett's Kingsley Hall poems, some of which I have referred to above. The archive mostly consists of personal essays, reflections on life at, or visits to, Kingsley Hall (and other PA communities). The contributions of the US psychiatrist Ross Speck, who visited in 1970, and Mike Yocum’s essay bear strong traces of the New Journalism, a term that came into wide circulation in 1973 with the publication of Tom Wolfe’s *The New Journalism.* In this genre, the personality of the writer is foregrounded, and character, setting and plot are presented with a fiction writer’s attention to detail. Humor, especially that which related to extreme situations, is often present. Not surprisingly, perhaps, the Hall's strangeness provided humorous material for Yocum and Speck. Yocum’s essay begins with his comic bafflement as someone opens the Hall front door to him with the words, “Do you have a frog to guide you?” (MS Laing L184/1-2). Yocum relates, too, a painfully funny story about giving a tranquilizer to a particularly annoying resident at the Archway community, a fellow resident who then becomes overly affectionate towards Yocum. Speck relates a conflict at the Hall dinner table during which he is drenched with wine. Followed out of the house by his assailant, Speck’s taxi driver asks, “Would you like me to punch him, Sir?” (MS Laing L179/1).

If Speck and Yocum adopted a literary form, they may have been influenced by Laing, who was celebrated for the evocativeness of his case histories and marketed as a literary figure by 1967 (Miller 2015). We can place him in a tradition of artists and writers interested in madness and altered states of consciousness—a tradition that goes back to the Romantics and on to Rimbaud’s 1871 injunction to undergo a systematic disordering of the senses to become a seer, a poet (2013). We might think, too, of the celebration of the madman we can find in Artaud (1958), or the Surrealists' interest in, and experiments with, states of madness and altered consciousness (Beveridge 2001). Laing was directly linked to radical literary traditions in the US and France through his friendship with Alexander Trocchi, the Beat writer who had lived in New York and who had been involved with the revolutionary artistic-political group, the Situationists, in Paris. In his "Book Proposal" for *Asylum*, Laing's style recalls that of a Beat poet, encouraging the reader to think of the Hall metaphorically, as a baby. The child, he writes, "was conceived by the union of desperate pain and daring vision." He continues:

The womb was a field of compassion extending back over many generations. The pregnancy was a complicated as you or I. It lasted many miles along a labyrinth, zigzagging under a farmhouse in Lincolnshire, through Tibet, Jerusalem, and Athens, to the Americas – via eternity and Western Europe. The delivery was in London and will happen when we look at how we stop ourselves from being and allow ourselves to feel the pains of old wounds. (MS Laing L259/90)

With its parallelism ("desperate pain and daring vision"), metaphor, and most of all with its driving energy (the movement across continents, "via eternity and Western Europe" and finally to London), the style is reminiscent of the US Beat poet Allen Ginsberg (whose most famous poem, *Howl*, Laing admired).

The *Asylum* documents not only demonstrate artistic presentation of the Hall but also reveal that some viewed the Hall itself as an artistic production. Both Speck and Musgrove present the Hall as a theatre, a spectacle defamiliarizing social roles and life's theatricality. Speck writes of Ian, a large man dressed as a woman, in the kitchen: “He imploded me as though I was walking in the middle of a Jean Genet play such as *The Blacks* or *Our Lady of the Flowers.*” Continuing, Speck writes, “My experience of these [sic] scene was more than a minor mind blower. My uneasiness immediately increased as I felt that an elaborately staged production was underway. I had the feeling I was to play a leading role as sacrificial scapegoat" (MS Laing L179/1). To enter into the spirit of the Hall, then, was to play a part. But Speck did not want to fulfill the part apparently "written" for him. Musgrove understands the Hall's theatricality in terms of Artaud's theatre of cruelty. “If I were to describe the community in terms other than its own, it would be as theatre. Not the kind in which a line is drawn between illusion and reality, but that described by Antonin Artaud as 'a call to conflicts; a subtle alchemy of magic and danger.' There was no stage and no proscenium, but an arena in which a pervasive self-consciousness blurred the customary distinctions between actor and audience, the player and his parts" (MS Laing L177/1). Musgrove and Speck's attention to roles and actor-audience relations may have been influenced by Irving Goffman, the author of the 1961 *Asylums.* In his *The Presentation of Self in Everyday Life,* he argues that the self is "performed" by actors on certain "stages" and in front of varying "audiences."

We can compare the Hall with its emphasis on performance and the breaking of boundaries to the central London Drury Lane Arts Lab London (1967-69). Laing and Hall residents visited the Arts Lab not as mere observers but as participants. The Lab, which focused on experimental arts, was a multi-media center containing a theatre, cinema, and gallery as well as a restaurant and a place for sleeping. The Lab inspired the establishment of similar ventures across the UK and was the forerunner of the still-running Institute of Contemporary Arts (ICA) in London. A key difference between the Lab and the ICA was that there was space at the Lab to "hang out" and "to crash." The intention was that there should be a dialectical relationship between everyday life and the arts with experimental living and experimental culture each drawing upon, challenging and re-making the other. We can compare this dialectic to the relation between Mary Barnes, her fellow residents, and her painting at the Hall. Her paintings allowed her to symbolize feelings that, before her beginning to paint, were largely unbearable and eluded representation. Her artwork affected others, some of whom responded positively, others negatively. Her art became a part not only of her recovery but also of the Hall's life.

Understanding the Hall as a single-building arts colony, then, does not preclude seeing it as a place of therapy. For Laing, madness, when fully experienced, *might* be creative, and madness played out in a supportive experimental community might be a fruitful and therapeutic response to alienation. Borrowing terms from Herbert Marcuse, a philosopher widely read by students and those identifying with the counterculture in the 1960s, the Hall, as a therapeutic art colony, posed an aesthetic challenge to the "one-dimensional" subject of post-war affluent capitalism (see Marcuse 1964, 1968). Pessimistic about organized working-class resistance, Marcuse saw hope, insofar as it existed, lying rather with marginalized groups and those who, in a utopian spirit, were moving beyond the dominant ideology. The Hall was an experiment that set out to enable people marginalized through their distress (and often through psychiatric diagnosis) to live through, and transcend, states of extreme disturbance, and the Hall was a place of creativity and creatively-inflected understandings of life.

It is difficult to define experimental art, but the literary critic Derek Attridge's recent definition is helpful. Such art is likely to be innovative, incompletely realized, a challenge to norms, bearing traces of its maker's trial and error procedures, difficult or perhaps impossible to categorize, with limited audience appeal, and falling outside the mainstream (2017). This sounds very much like the Hall. The community was innovative; it was a project, too, that ended after five years and was not fully "written up." The *Asylum* project contains texts that attest to "trial and error" procedures. The community was many-faced, difficult to define and, while it attracted much countercultural and indeed mainstream media interest, remained strange to the psychiatric mainstream.

## A countercultural experiment with the accent on therapy as attention to the practice of everyday life

In this section, I want to explore further ways in which the Hall was an experiment. For Zeal in his account of life at the Hall, it was somewhere “in the midst of daily life, not at its periphery, [that] I could experiment with others in being free” (MS Laing L218). He is not the only contributor to the book who writes of the community as an “experiment.” In part, the experiment was concerned with trying to establish what I have termed an inner-spaceship concerned with constructing a kind of countercultural community, one that for several residents had spiritual and artistic dimensions, which sat uneasily with its East End neighbors. Cultural production, spirituality, community, and inner space exploration were bound up with therapy, not therapy in a formal sense but rather therapy as a practice ingrained in everyday life. We can think further about the Hall's therapeutic character by examining the scientific, medical and social scientific meanings of experimentation to be found in *Asylum*, as well as countercultural usages of the word that associate the Hall with" experimental lifestyles (with "being free" in Zeal's words).

We find the scientific-medical *and* countercultural meanings in a piece by Leon Redler, *Asylum's* editor, for whom the Hall is “an experimental residential community.” He writes: “We wanted to observe the course of lives of people diagnosable as schizophrenic, schizoid or otherwise mentally ill when allowed to live without the implicit invalidation and stigmatization of the psychiatric diagnosis and without the physical, social and biochemical restraints of institutional psychiatric treatment.” The "we" consists of observers, presumably medically legitimated professionals. Redler sets out a hypothesis: “we wanted to see if and under what conditions acute mental breakdowns could be followed by healing and/or renewal and whether long-term mental and social impasses could be alleviated in a non-institutional and potentially creative living situation.” Here the Hall sounds for the most part like a social science or medical research project, but the words that follow undermine this view and offer poetic and spiritual images of the community. Redler says the Hall was to be “A lifeboat, a refugee ship, heading for unmapped seas [….] A ship of fools in a sea of samsara, risking much for the possibility of discovering, together, a way home” (MS Laing 221/15A). The project's aim was to provide a homecoming, "home" here suggesting (in a way characteristic of the counterculture) a return to an originally authentic state, which would afford release from the sequence of birth and death (samsara). Redler, then, faces two ways: towards medical professionals and others in the caring professions and the academy, and towards those in the underground for whom experimental living offers the homecoming of authenticity.

Redler's juxtaposing of mainstream research with experimental living suggests that while the Hall was indeed a countercultural project, we should not see it as a community cut off *entirely* from mainstream psychiatry. Oisin Wall (2017) reminds us that the PA retained links with mainstream psychiatry, with, for instance, the pioneer of the therapeutic community movement, Maxwell Jones, acting as a PA patron. And in *Groovy Science,* W. David Kaiser and Patrick McCray (2016) challenge the common assumption, derived in large measure from Theodore Roszak (1969), that the counterculture was fundamentally anti-science. They argue that the relation between “straight,” mainstream science and the counterculture was rather more complicated with the two spheres sometimes being far from separate and distinct. Indeed, I would add that the two came together in SOMA, a research institute founded by Stephen Abrams, who had conducted ESP experiments at Oxford, organized the full page 1967 *Times* newspaper call for cannabis legalization, and helped establish Release, an underground initiative that supported people arrested by the police. SOMA's aim was to investigate the effects of hallucinogens. The institute's advisory board included Laing and Cooper, as well as figures of unimpeachable "straight" science credentials, such as Francis Crick.

Just as Redler's *Asylum* introduction gestures towards two audiences—on the one hand, the medical and academic and on the other hand, a broader readership interested in, or identifying with, the counterculture—so the US psychiatrist Ross Speck's two contributions look two ways. His first is a highly descriptive narrative essay indebted to the New Journalism. "Kingsley Hall: A Metaphor" presents his 1970 Easter Sunday visit to the Hall (MS Laing L179/1). A second essay, however, adopts a scholarly tone. In "What You See is Where You're At," Speck writes of his own perspective having been formed by his own background, presumptions and values (Uncatalogued). If we understand the two essays in relation to one another, Speck's work, combining narrative and reflexivity, fictive techniques and epistemological claims, fits easily into the "blurred genre" of autoethnography that can be found especially in Communication Studies, Sociology, and Education. (Ellis, Adams, and Bochner 2010). He combines sympathy for the Hall project with an awareness of his own difficulty in writing about it. He finds the ease with which residents can move between roles (the intellectual, the professional, and the patient) very impressive. But the community, he says, is an "in-group," and it is difficult for an outsider to enter and accurately write about it: "The formation of a group and its accompanying ideology is accomplished by hard work and inner turmoil, complete with initiation, dues, and rites. The intruder reaps (rapes) some of the group’s treasures without paying this price" (Uncatalogued). Given the outsider-researcher's distance from the object of research, despite being committed to the project, he or she inevitably errs. At worst, Speck points out, the researcher runs the risk, emphasized also by his colleague Carolyn Attneave in her *Asylum* contribution (Uncatalogued), of being like a white anthropologist misrepresenting a native American tribe.

Speck's concerns recall the counterculture's suspicion of outsider representations of itself, concerns epitomized by one of the underground's favorite artists, Bob Dylan. His "Ballad of a Thin Man" addresses an outsider, Mister Jones, a writer who tries but inevitably fails to understand "hip" scenes. "You put your eyes in your pocket and your nose on the ground," sings Dylan. "Something is happening but you don't know what it is / Do you, Mister Jones?" (2018). His problem is his "stance"; with his nose to the ground, he cannot sniff out the truth, and with his eyes in his pocket, he cannot see what is before him. What he writes, then, can only be a misrepresentation. A similar concern with "straight" world misrepresentation is present in a satirical article entitled "Stereophenia" that appeared in *Friends*, the UK underground newspaper (UCL Special Collections, LITTLE MAGAZINES FRI, March 24, 1970).The article is presented in sections that parody a scientific article: The Problem (a music-related disease characteristic of technologically advanced societies); The Symptoms ("blown," "high" minds and involuntary "grooving" when listening to rock music); The Project (initiated by The Department for Environmental Manipulation); The Experiment (two groups were observed, one given and one denied drugs and music); The Results (mixed, with some "groovers" having little dependency on equipment and some spontaneously climaxing); and Towards Treatment (centers for addicts and the threat of removing records from circulation). The article is accompanied by a photo of two young men sitting among records and drug paraphernalia. The argument is clear: psychiatry and medicine more generally, do not—and cannot— understand the underground so far as they observe rather than participate.

The US psychiatrist, Loren Mosher, who visited the Hall and later went on to establish Soteria House (a therapeutic community in San Jose, California), proudly signals his outsider status and distance from the counterculture in his *Asylum* contribution's title: "Kingsley Hall as Experienced by a Member of The American Establishment." He presents himself amusingly as an enthusiastic young researcher keen to collaborate with Laing or perhaps just receive his blessing for a project. But when the two meet, Mosher is disappointed. He leaves Laing “with a bladder full of the excretion products of several cups of tea, a notebook with names of other people involved in the “network,” and no program whatsoever.” Mosher wanted to scrutinize the Hall in a professionally valued form with findings appealing to the psychiatric profession. He thought the Hall experiment valuable and transposable to other places but that “The model need not be anti-establishment.” Mosher continues: "Kingsley Hall prompted me to run a ward in a Community Mental Health Center based on them and later to develop a careful research design to evaluate, in the logical positivist sense, the effectiveness of this kind of setting in the USA" (MS Laing L173/4). His reference to positivism refers presumably to measurement, independent verification and statistics (rather than, as he puts it, "logical positivism"). Published in a psychiatric journal, his research, co-written with Alma Menn, into Soteria House, a treatment facility staffed largely by trained non-medical carers looking after first-time diagnosed schizophrenics, claimed positive results*.* Research subjects, selected from a community mental health center, were contrasted with a "control" group from the same source and sent to a psychiatric ward. Patients completed a battery of psychological tests and self-report questionnaires. Exercising "considerable caution" because his research sample was so small, Mosher claimed that results indicated that "the need of hospitalization can be treated successfully by a nonprofessional staff, usually without medication, at no greater cost, in a home in the community” (1979, 83). While it would be unfair to view him merely as a Mister Jones figure or a doctor who might subscribe to the "stereophenia" diagnosis, Mosher's research looks nothing like anything in *Asylum*—despite his discomfort with the term "schizophrenia" and his belief that psychosis can be a growth experience*.*

Laing moved away from mainstream psychiatric publication. However, he had begun his career as a medical author by co-writing an article in *The Lancet* about the Rumpus Room, a room in Gartnaval Hospital, Glasgow, in which selected female refractory ward patients gathered each day and were able to cook, sew, make tea and chat (Cameron, Laing, and McGhie 1955). Their condition, the authors claimed, improved significantly. Although without the psychological tests and scales of Mosher's work, the Rumpus room article is more like Mosher's research than the work in *Asylum.* But Laing's understanding of research changed. His major research project at London's Tavistock Institute involved work with his fellow psychiatrist Aaron Esterson. Esterson interviewed female schizophrenics and their families, and Laing wrote up the research in their *Sanity, Madness and the Family.* The book argues that what is usually diagnosed as schizophrenia can be better understood as an attribution made by medical professionals to behavior that is intelligible in terms of the position the supposedly mad person occupies in a family network. Laing's manner of writing is far from the rhetoric of the psychiatric case study or a text that might find publication in an academic journal. Aside from the presentation of interviews with eleven families—eleven families of "schizophrenic’ daughters"—Laing and Esterson offer only a brief presentation of the researcher’s fundamental research question together with a scanty outline of methodology. Chapters do not end with any analysis; there is no discussion of findings or conclusion at the close of the text. The text is quite literary in flavor—and inspired Hilary Mantel (2008), now one of Britain's leading authors, to begin writing fiction. In Laing's presentation of the family interviews (which are not given verbatim), we find subterfuge, deception and painfully humorous ironies and misunderstandings. The text implies an author whose vision is literary and satirical and strongly critical of the restrictive lower middle-class morality prevailing in the schizophrenic girls' families. Above all, the author is someone who has no investment in neutral observation; he is rather on the side of the person labeled "schizophrenic." *Sanity, Madness and the Family* signals Laing's move away from the psychiatric mainstream, as, more clearly, does the later *Politics of Experience*, which was rejected by its first medical reviewer and accepted by a second reviewer because of its literary qualities (see Miller 2015). Not only did Laing, by the time of Kingsley Hall, have no desire to publish in academically or medically valorized forms nor did most who subscribed to the Hall project. But the Hall was a research project all the same, and one that was at least in part written up in Mary Barnes' and Joseph Berke's *Two Accounts*. Evidence comes largely in the form of first-person testimonies, and by far the longest and most detailed sections are those not of Doctor Berke but of Barnes who underwent the “journey” through madness. The *Asylum* book, had it come to fruition, would have presented a further writing up of Hall research and would have included Mosher’s text criticizing Laing and the Hall. Like *Two Accounts*, the book would have been aimed not at an exclusively professional or academic audience but rather at a much broader public.

Laing was opposed to what he terms, in *The Voice of Experience's* opening chapter "The Objective Look," a way of researching nature or human beings that in his view detaches the observer not only from the object of research but also from his or her own experience. What is more, Laing argues that the objective look requires questions of morality and value to be put aside, along with “joy and sorrow, misery and happiness, pleasure and pain, right and wrong” (1982, 34). Experience, including inklings of one's birth or even pre-birth states or paranormal states, must be disregarded as *a priori* unscientific. While informed very much by his understanding of phenomenology, Laing's "objective look" is very similar to Theodore Roszak's "objective consciousness," a way of doing science that, he claims, results in a de-sacralization of nature. But Roszak came to see science as having a *possible* playful and heuristic dimension. This brings to mind a specific countercultural experiment: the London Art Lab. Writing about the Drury Lane Arts Lab, the theatre critic Kenneth Tynan wrote that "Like any good lab, it sometimes houses experiments that blow up in their investors’ faces; but it’s blessedly unsmart, and you get a happy sense of work-in-progress, of new departures that may blossom into new arrivals" (2018). An observer sympathetic to the Hall may well have viewed it similarly.

For Laing, certainly, observation had to go hand in hand with participation. The Laing archive contains correspondence between Noel Cobb and Laing before Cobb's arrival at the Hall. Cobb, who had been living in Norway, wrote to Laing explaining his interest in radical psychiatry and asking if Laing could offer him some work. Replying, Laing states that he cannot offer much money but it might be possible for Cobb to receive a small sum for working as a “social therapist” in and around Kingsley Hall and for engaging in what Laing calls “our work with LSD.” Laing concludes: “I think it could only be a question of coming to London and discovering once here the possibilities of joining in the dance” (MS Laing GC 147). Kingsley Hall here, then, is not an ordinary therapeutic household or standard research project; it is a “dance.” Valuable involvement in the countercultural living laboratory requires physical engagement, commitment, and fellow feeling. It is not a place, then, for a detached observer, for someone who does not see him- or herself as a partner in the project or who does not “swing” with it in an embodied fashion. This view clarifies the non-meeting of minds between Laing and Mosher. The point, for Laing, was not to establish by the use of tests or scales that the Hall was an effective means of treatment. The Hall was effective for those who wholeheartedly subscribed to the project. The community represents Laing’s attempt to transcend technocratic expertise and to engage in research as a participatory dance, thereby investing experimentation with personal engagement and transcending divisions between researcher and research subject.

What most discomforted Mosher about the Hall was what he viewed as a sort of adolescent nose-thumbing at everyday mores: “It was as though introductions, hand shakings and get-acquainted small talk were somehow *proscribed* [*…*] my uncertainty about how welcome I was there remained throughout the year,” he writes (MS Laing L173/4). This view contrasts with that of George Spencer-Brown, a mathematician and writer, and author of *The Laws of Form*, who lived at the Hall. For Spencer-Brown, the rule at Kingsley Hall—the nature of the experiment, we could say—was indeed to break everyday rules, but what was in play was far more profound than the transgression of everyday niceties. The aim was, he says, “to free ourselves from not what might be called conventions of life (conventions I take not to be what are actually consciously called into being as rules such as you don’t eat peas with a knife), but rather the un-written, un-conscious arrangements which are, as it were, programmed into us from very early years and which we have forgotten exist." He gives an example, a dinner-time discussion when Laing asked everyone in turn how they thought they were going to die. It was not enough to answer “cancer.” Laing would probe: “yes, cancer of where?” After his initial horror, Spencer-Brown claims that he and other residents found value in this style of boundary-breaking. He continues: "the rule was that we shouldn't try and hide our feelings from each other, that we should be as direct as possible. In other words, in ordinary social parlance, that we should be as rude to each other as possible. Rude as in its original meaning, not impolite but as primitive-direct" (MS Laing L191/1-2). What Mosher understands as bad manners was to Spencer-Brown rather a sign of what seems like an informally constituted encounter group, a social situation in which participants must, despite their discomfort, reveal their most fundamental fears and desires (with this leading to greater authenticity, which is itself a return to the primitive, a state prior to the excrescences of civilization). Such "encounters" may have defamiliarized—that is, made strange—"un-written, un-conscious arrangements" underpinning societally sanctioned existence, thus introducing the possibility of change. What was adolescent truculence to Mosher, then, could be viewed (through Spenser-Brown's lens) as essential to the Hall's style of experimental, countercultural living—the attempt to be free.

Nick Crossley (1999), a sociologist for whom the Hall was a "Working Utopia" (WU), offers an additional way of understanding the household's challenge to everyday mores. WUs are sites that offer social movements the possibility of experimentation, research, and movement expansion. A WU is also a site of "pedagogic action" (Crossley 1999, 817-819). Embodied habits, presuppositions and ordinary ways of acting and relating to others—what Crossley, borrowingBourdieu's term, refers to as "habitus"—are challenged. This is just how Spencer-Brown experienced Kingsley Hall. For Crossley, whose brief comments on the community rely on published sources, the Hall's pedagogic action was bound up with formal modes of education (e.g. meetings, lectures, seminars). Such events took place, but Spencer Brown's testimony points to a broader experience of education, a kind of therapeutic learning ingrained in everyday life. Yocum presents a similar position in his essay. Reflecting on the value of living in PA houses, he says, “No longer did the fact that someone spoke my own language mean that his speech would be understandable. No longer, in general, could experience be relied upon a guide for the present. Nothing could be taken for granted. Nothing assumed.” And this he found highly valuable. He was jolted into seeing the world anew; presumptions became apparent; the effect was “unnerving but enlivening” (MS Laing L185/1-2).

## Conclusion

There were numerous Kingsley Halls; people experienced it from varying standpoints. As Laing put it in a discussion with Redler and Sid Briskin, a social worker involved in setting up the Hall: "one of the problems of recounting this story is that the linear form of verbal narrative is unadapted to the reality of the events, which are a set of patterns that undergo transformations, involving many people" (quoted in Harris 2012, epigraph). Laing highlights the problem of representing the Hall. The *Asylum* book, consisting of various accounts in various genres would have given us "sets of patterns" and "transformations, involving many people." With varying viewpoints, given in various forms, the book would have offered a patchwork view of the Hall, with one voice interrupting, sometimes confirming and sometimes contradicting the previous one. Even in its incomplete state, *Asylum* offers a rich sense of what the Hall meant to several residents.

This discussion of accounts has drawn out a number of themes, each of which could be elaborated in further research. The ways of thinking about the Hall set out here explore the nature of the experiment and supplement our understanding of Laingian psychiatry in terms of counterculture (Chapman 2015; Wall 2017). Like the work of Franco Basaglia and the Italian *Psichiatria Democratica* movement, the residents of Kingsley Hall were 'doing 1968' before the revolutionary year of '68. What is more, with the community's focus on inner space, its interest in spiritual renewal and development, and its emphasis on culture—all adding up to a countercultural experiment with a close focus on the liberating potential of therapeutic mutual support—the Hall exemplifies some key aspects of radical counterculture in the long 1960s. Indeed, the Hall experiment was so clearly countercultural that it is quite appropriate to term this phase of Laing’s work “countercultural psychiatry” (a term that specifies the Hall’s version of radical psychiatry). Future work may further explore the Hall’s relation to other countercultural forms of therapy (e.g. encounter groups).

Clearly, the community was bound up with numerous other projects in alternative London and beyond. It was part, as I have pointed out, of an international movement. In Berke’s edited 1969 volume *Counter Culture*, there is an essay on the Hall alongside material on communes, arts labs, alternative cinema, revolutionary politics and theatre and how to leave cheaply on the margins of society. The book features content from the UK, mainland Europe and the USA. *Counter Culture’s* subtitle is *The Creation of an Alternative Society.* We can view the Hall as an attempt to “build the new world,” the alternative society, “in the shell of the old world” (to employ the idiom of the Wobblies, the US International Workers' of the World trade union). Seen this way, we can place the Hall, a project in the politics of prefiguration—an attempt, that is, to give a taste in the present of how the future could be—in the tradition of anarchist and libertarian projects in which progressive initiatives seek to *enact*, as far as possible, a model of the good society (Boggs 1977; Breines 1982; Cornish et al. 2016).

But can we relate the Hall to the present? Recently Peter May, a widely-selling crime writer, has employed a version of the Hall (named "Victoria Hall" and placed in East London's Bethnal Green) to present, in miniature, 1960s London and the period of countercultural experimentation and excitement more broadly. In *Runaway* (2015)*,* May makes a contrast between, on the one hand, the social solidarity, daring and hopefulness of the radical 1960s, symbolized by his version of the Hall, and, on the other hand, "atomized" neoliberal subjectivity, symbolized in the novel by a young man called Ricky who spends most of his time playing computer games alone and requires the intervention of his grandfather to leave his bedroom and start imagining another future for himself. In the twenty-first century, could Kingsley Hall help us imagine another future? Laing envisioned a near-future of Hall-like communities in urban and rural locations. These would be places where people who had become disturbed and needed sanctuary might go, perhaps for a brief stay, perhaps for a long time (MS Laing A 537/1-2). That such a broad network of communities never came into existence might suggest that the original model was flawed. Certainly, there were problems at the Hall: it was not a place for everyone who was in distress and was not somewhere for everyone who was interested in helping others in distress. The community was seriously fractured from its neighbors. To benefit from the project, one had to "sign up" to its values. And, as I have explored elsewhere (Chapman 2020), the aim of dissolving the doctor-patient binary was realized only partially. But this essay reveals a wide range of testimonies concerning the Hall that, like Barnes' and Berke's, are positive. The accounts imply that the community should be understood as a significant experiment in the tradition of liberatory psychiatry. If the Hall did not inaugurate a much wider movement, I suggest that this was not so much because of its failures but rather because the counterculture, of which it was very much a part, was politically defeated. The material preconditions for widespread oppositional counterculture—relatively cheap housing, widespread secure employment, student grants, adequate welfare payments—no longer exist. Now, however, there is some recognition on the Left that the counterculture of the 1960s and 1970s might be drawn upon by a twenty-first century politics that points beyond neoliberal hegemony.

In a gesture towards Laing, who was referred to as an "acid Marxist," Mark Fisher (2009, 2019), who authored a critique of what he understood as restrictive and illusory neoliberal "common sense," which he called *Capitalist Realism*, began to write a book entitled *Acid Communism*, a consideration in part of how radical cultural practices associated with the 1960s and 1970s might inform present-day politics. Rejecting the view that counterculture essentially led to the neoliberal "trap" of consumerism and neoliberal hegemony (Curtis 2007; Žižek 2008), acid communism—which does not require the use of psychedelics—sees countercultural experimentation as beneficial to, if certainly not alone sufficient for, for the Left. Utopian projects at the margins of capitalism's possibilities may gesture towards, and indeed partially or inconsistently inhabit, another world. Fisher, like the cultural and political theorist Jeremy Gilbert (2017), who has further elaborated the idea of acid communism (or "psychedelic socialism" as it is sometimes called), had little knowledge of or time for Laing. Yet the accounts of Hall life considered here exemplify several traits of acid communism as set out by Gilbert: social solidarity, playfulness and adventure, the exploration of what it means to be free, plus the conscious, collective attempt to remake everyday life beyond restrictive and conventional mores. It was no accident that Leon Redler, the editor of *Asylum: To Dwell in Strangeness*, addressed meetings at Occupy London (2011-2012), the protest camp outside St Paul's cathedral (Personal Communication). In common with Occupy groups in New York and elsewhere, Occupy London sought to draw attention to inequality and global injustice, and to enact (through horizontal decision-making and openness to diversity) a vision in the present of a more progressive world. Redler sensed a common thread between the Hall and Occupy. That thread may yet be sewn into radical projects (in mental health and beyond) in time to come.

## Endnotes

^1^Call numbers, each beginning "MS Laing", are given for archival sources in the R. D. Laing Collection, Archives and Special Collections, University of Glasgow Library.
