# Correction to: Dwelling in Strangeness: Accounts of the Kingsley Hall Community, London (1965-1970), Established by R. D. Laing

**DOI:** 10.1007/s10912-021-09685-3

**Published:** 2021-03-13

**Authors:** Adrian Chapman

**Affiliations:** Florida State University London Centre, 99 Great Russell Street, London, WC1B 3LH UK

**Correction to: Journal of Medical Humanities**

**https://doi.org/10.1007/s10912-020-09656-0**

Keith Musgrove’s surname was incorrectly spelled as “Musgrave” in the article. The author would like to apologize for the error.

The original article has been corrected.

